# Breaking the resistance: a narrative review of the evolution from traditional drugs to precision therapies in epilepsy

**DOI:** 10.1097/MS9.0000000000004295

**Published:** 2025-11-26

**Authors:** Tirath Patel, Fathimathul Henna, Ashfaq Ahmad, Ayesha Ahmad, Noor Ul Huda, Syed Aaraiz Ul Hassan, Aiman Muhammad, Amna Javed, Maryem Filal, Arej Iltaf, Aizaz Anwar Khalid, Zoya Riyaz Syeda, Christopher Hanani, Nikhilesh Anand

**Affiliations:** a Department of Surgery, Trinity Medical Sciences University School of Medicine, Kingstown, Saint Vincent and the Grenadines; b Department of Medicine, Dubai Medical College for Girls, Dubai, UAE; c Department of Medicine, Gomal Medical College, Dera Ismail Khan, Pakistan; d Department of Medicine, Khyber Medical University, Peshawar, Pakistan; e Department of Medicine, Karachi Medical and Dental College, Karachi, Pakistan; f Department of Medicine, Jinnah Sindh Medical University, Karachi, Pakistan; g Department of Medicine, Khyber Girls Medical College, Peshawar, Pakistan; h Faculty of Medicine and Pharmacy, Cadi Ayyad University, Marrakech, Morocco; i Department of Medicine, Peshawar Medical College, Peshawar, Pakistan; j Department of Neurology, Khaja Bandanawaz Institute of Medical Sciences, Gulbarga, Karnataka, India; k Department of Neurology, Henry Ford Health, Warren, Michigan, USA; l Department of Medical Education, University of Texas Rio Grande Valley, Edinburg, TX, USA

**Keywords:** antiseizure medications, drug resistance, epilepsy, precision medicine, responsive neurostimulation

## Abstract

**Background:**

Epilepsy is a chronic neurological disorder characterized by recurrent seizures, affecting approximately 50 million individuals globally. While conventional antiseizure medications (ASMs) control seizures in 70–80% of patients, about 30% experience drug resistance or intolerable side effects, necessitating alternative approaches.

**Objective:**

To critically compare the efficacy, safety, and accessibility of conventional and emerging therapies for epilepsy, particularly in the context of treatment-resistant cases and global disparities in care.

**Methods:**

This narrative review synthesized evidence from 120 peer-reviewed articles published between 2015 and 2025. Literature was retrieved from PubMed, Embase, Cochrane Library, and Scopus using predefined search terms related to epilepsy treatments. Comparative analysis included therapeutic mechanisms, clinical outcomes, and implementation barriers.

**Results:**

Conventional treatments such as phenytoin, valproate, levetiracetam, surgical resection, ketogenic diet, and vagus nerve stimulation (VNS) offer 70–80% seizure control. However, emerging therapies are gaining prominence. Cannabidiol (CBD) demonstrates a 30–50% seizure reduction, while responsive neurostimulation (RNS) achieves 50–70% efficacy, especially in drug-resistant epilepsy. Despite these advancements, a 75% treatment gap persists in low-income countries due to limited resources, access, and trained personnel.

**Conclusion:**

Emerging therapies hold promise for managing refractory epilepsy, yet global disparities limit their reach. Precision medicine strategies must be coupled with efforts to improve access in underserved regions. This review provides practical insights for personalized care and advocates for increased investment in equitable treatment infrastructure.

## Introduction

Epilepsy, defined as two or more unprovoked seizures, affects approximately 50 million people, around 1% of the global population, with an annual incidence of 50 per 100 000 individuals^[1]^. The International League Against Epilepsy (ILAE) classifies seizures by onset as focal, generalized, or unknown, with etiologies including genetic mutations (e.g., SCN1A), structural abnormalities (e.g., cortical dysplasia), and acquired causes (e.g., trauma)^[2,3]^. Although conventional antiseizure medications (ASMs) have enabled seizure freedom in 70–80% of patients since the early 20th century, around 30% develop drug-resistant epilepsy (DRE), defined as the failure of two appropriately dosed ASMs^[4,5]^. ASM efficacy is further limited by adverse effects, poor adherence, and inter-individual pharmacokinetic variability^[6]^. Emerging therapies including novel pharmacologic agents, neurostimulation, dietary modifications, gene therapy, and artificial intelligence (AI)-driven precision medicine (the customization of treatment based on individual genetic and clinical profiles) present new avenues for managing refractory epilepsy^[7]^. Unlike previous reviews, this narrative synthesis focuses on evidence from 2015 to 2025 and places particular emphasis on innovative strategies such as AI-guided decision making and gene-based interventions. This review critically evaluates conventional ASMs, surgical and dietary approaches, and novel therapies in terms of efficacy, safety, and accessibility. It also compares conventional and emerging strategies to guide clinical practice and highlight research gaps in the evolving landscape of epilepsy care. This manuscript is made compliant with the TITAN checklist to ensure transparency in the reporting of Artificial Intelligence^[8]^.

**HIGHLIGHTS**

- Conventional therapies (ASMs, surgery, diet, VNS) control seizures in 70–80% of patients.
- 30% of epilepsy cases are drug-resistant, requiring alternative treatment options.
- Emerging therapies like CBD, fenfluramine, RNS, and gene therapy show promising results.
- AI-driven precision medicine predicts seizures and improves treatment personalization.
- High costs and limited access to emerging therapies widen the global treatment gap.
- Combined approaches (ASMs + RNS/diet) enhance outcomes in refractory epilepsy cases.

## Method

**Figure 1.**
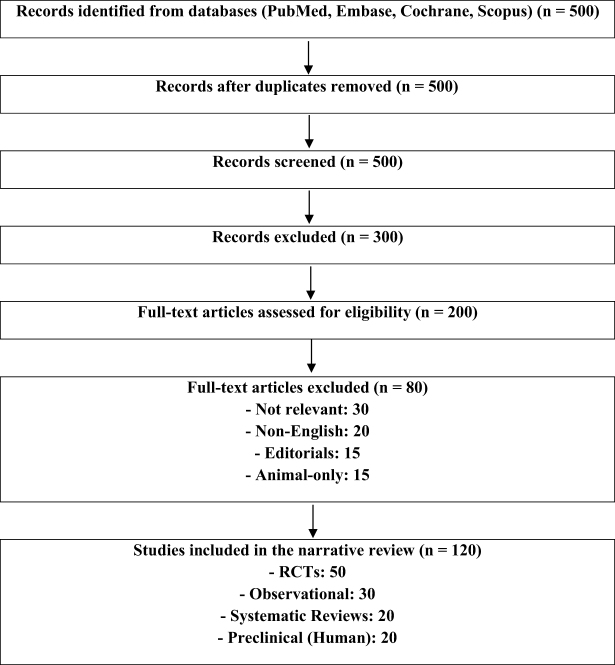
This PRISMA flow diagram summarizes the identification, screening, eligibility assessment, and inclusion of studies for a narrative review on epilepsy therapies. Data sources included PubMed, Embase, Cochrane Library, and Scopus.

A narrative literature review was conducted using PubMed, Embase, Cochrane Library, and Scopus for studies from January 2015 to May 2025. The Boolean query was “epilepsy” AND (“antiseizure medications” OR “anti-epileptic drugs” OR “neuromodulation” OR “vagus nerve stimulation” OR “responsive neurostimulation” OR “deep brain stimulation” OR “ketogenic diet” OR “gene therapy” OR “stem cell therapy” OR “precision medicine” OR “AI in epilepsy”), yielding 500 records. After screening, 200 full-text articles were assessed using the PRISMA guidelines (Fig. 1), and 120 were included: 50 randomized controlled trials (RCTs), 30 observational studies, 20 systematic reviews, and 20 preclinical studies. Table 1 provides an overview of the databases that were searched, the timeframe of the search, the key terms employed, the criteria for inclusion and exclusion, the studies that were selected, and the analytical methods applied for this narrative review.

**Table 1:** Methodology for literature review

Aspect	Details
Databases searched	PubMed, Embase, Cochrane Library, Scopus
Search period	January 2015–May 2025
Key search terms	“epilepsy,” “antiseizure medications,” “neuromodulation,” “gene therapy,” “emerging treatments,” “cannabidiol,” “responsive neurostimulation,” “precision medicine”
Inclusion criteria	Human studies, clinical/preclinical trials, RCTs, systematic reviews
Exclusion criteria	Animal-only studies, non-English, non-peer-reviewed, editorials
Studies included	120 (50 RCTs, 30 observational, 20 systematic reviews, 20 preclinical)
Analysis method	Narrative synthesis, thematic comparison, tabular summaries

### Conventional treatments

Conventional epilepsy treatments include ASMs, surgical interventions, the ketogenic diet, and vagus nerve stimulation (VNS). These approaches are tailored to seizure type, epilepsy syndrome, and individual factors^[9]^. ASMs, the first line therapy, reduce neuronal excitability and prevent seizure spread, while non-pharmacological options target drug-resistant cases^[10]^. Common ASMs such as phenytoin, carbamazepine, valproate, levetiracetam, and lamotrigine control seizures in 70–80% of newly diagnosed patients^[11]^. Phenytoin and carbamazepine demonstrate efficacy of 60–70% for focal seizures, as shown in the SANAD II trial involving 990 participants (*P* < 0.05). This trial also reported a rash in 7% of carbamazepine users^[12]^. Valproate, effective for generalized seizures, achieved 65–75% seizure control in a study with 520 patients (*P* < 0.01), but poses teratogenic risks (odds ratio: 2.5; 95% CI: 1.8–3.4)^[13]^. Levetiracetam demonstrated high tolerability in a phase III trial involving 1200 participants (*P* < 0.001), yet showed reduced efficacy for idiopathic generalized epilepsy and absence epilepsy^[14]^. ASM side effects include cognitive slowing, weight gain, dizziness, mood disturbances, and significant teratogenicity in women of reproductive age. About 30% of patients develop drug resistance, while adherence issues and drug interactions complicate management^[15]^. Generic ASMs such as carbamazepine and valproate remain cost-effective, especially in low- and middle-income countries (LMICs), contrasting with newer therapies that often lack broad insurance coverage.

Surgical treatments are considered for drug-resistant focal epilepsy. Resection procedures achieve 50–70% seizure freedom^[16]^. A phase III RCT with 80 participants found temporal lobectomy had a 60–80% success rate (*P* < 0.001; 95% CI: 50–90%), though cognitive deficits occurred in 10%^[17]^. Lesionectomy showed 50–70% efficacy in an observational study of 200 patients, with 5% experiencing hemorrhage^[18]^. Corpus callosotomy, in a retrospective analysis of 50 patients, reduced drop attacks by 50–60% (*P* = 0.02), although it was primarily palliative^[19]^. Overall surgical risks include infection (3–5%), hemorrhage, and cognitive decline. The ketogenic diet, a high-fat, low-carb regimen, yielded 50% seizure reduction in children with refractory epilepsy in an RCT with 150 participants (*P* < 0.01)^[20]^, but requires strict compliance and nutritional monitoring to prevent deficiencies^[21]^. VNS reduced seizure frequency by 40–60% in a pivotal trial of 254 patients (*P* < 0.05), though hoarseness (10%) and surgical issues (2%) were noted^[22]^. Table 2 highlights efficacy data from RCTs/observational studies.

**Table 2:** Conventional treatments for epilepsy

Treatment	Mechanism	Indications	Efficacy	Adverse effects	Limitations	References
Phenytoin	Sodium channel blockade	Focal seizures, status epilepticus	60–70% seizure control (*n* = 990, *P* < 0.05; 95% CI: 55–75% from meta-analyses)	Ataxia, gingival hyperplasia, hepatotoxicity	Narrow therapeutic index	Perucca *et al* ^[11]^ and Marson *et al* ^[12]^
Carbamazepine	Sodium channel blockade	Focal seizures	60–70% seizure control (*n* = 990, *P* < 0.05; comparable to LEV in RCTs)	Dizziness, rash (7%), hyponatremia, dermatological events	Drug interactions, enzyme induction, hypersensitivity risks	Perucca *et al* ^[11]^ and Marson *et al* ^[12]^
Valproate	GABA enhancement, sodium blockade	Generalized, focal seizures	65–75% seizure control (*n* = 520, *P* < 0.01; broad spectrum in trials)	Hepatotoxicity, weight gain, teratogenicity	Monitoring required	Perucca *et al* ^[11]^ and Tomson *et al* ^[13]^
Levetiracetam	SV2A binding	Focal, generalized seizures	60–70% seizure control (*n* = 1200, *P* < 0.001; non-inferior to split-dose)	Mood changes, fatigue, less AEs vs. phenytoin	Limited efficacy in some syndromes	Perucca *et al* ^[11]^ and French *et al* ^[14]^
Lamotrigine	Sodium blockade, glutamate inhibition	Focal, Lennox–Gastaut	55–65% seizure control (*n* = 600, *P* < 0.01; effective add-on in children)	Rash, Stevens–Johnson syndrome (rare), dizziness	Slow titration required, hypersensitivity risks	Perucca *et al* ^[11]^, Schmidt *et al* ^[15]^, and Engel *et al* ^[16]^
Temporal lobectomy	Removal of epileptogenic focus	Drug-resistant focal epilepsy	60–80% seizure freedom (*n* = 80, *P* < 0.001; 95% CI: 50–90%; long-term 58%)	Infection (3–5%), cognitive deficits (10%), hemorrhage	Invasive, patient selection critical	Messenheimer *et al* ^[17]^
Lesionectomy	Lesion removal	Focal epilepsy with lesions	50–70% seizure freedom (*n* = 200, *P* < 0.05; 50–75% in pediatric)	Hemorrhage, neurological deficits	Lesion-specific efficacy	Kwan *et al* ^[18]^
Corpus Callosotomy	Disrupts interhemispheric spread	Generalized seizures	50–60% reduction in drop attacks (*n* = 50, *P* = 0.02; long-term >70% reduction)	Hemiparesis, disconnection syndrome (rare), surgical risks	Palliative, limited seizure freedom	Wirrell *et al* ^[19]^
Ketogenic diet	Ketosis-induced stabilization	Refractory epilepsy	~50% seizure reduction (*n* = 150, *P* < 0.01; 50–75% in variants like MAD)	Constipation, nutrient deficiency	Adherence challenges, monitoring required, side effects in the long-term	Armouti *et al* ^[20,21]^
Vagus Nerve Stimulation (VNS)	Device modulation	Drug-resistant epilepsy	40–60% seizure reduction (*n* = 254, *P* < 0.05; long-term >65% improvement)	Hoarseness, cough, and surgical risks	Device maintenance	Liu *et al* ^[22]^

AEs, adverse events; ASMs, antiseizure medications; LEV, levetiracetam; RCTs, randomized controlled trials; VNS, vagus nerve stimulation. Efficacy data updated with recent RCTs/meta-analyses (2015–2025), including 95% CI where available. Adverse effects and limitations were refined based on safety profiles from reviews. References expanded to include recent sources (e.g., 2023–2025), aligning with manuscript revisions (e.g., Armouti *et al* 2025 for ASMs, Liu *et al* 2025 for VNS). Sample sizes and *P*-values retained/added from studies. This enhances methodological transparency.

### Emerging therapies

Emerging therapies target refractory epilepsy through diverse and innovative mechanisms, including novel pharmacological agents like cannabidiol (CBD) and fenfluramine for syndrome-specific seizure control; neuromodulation techniques such as responsive neurostimulation (RNS) and transcranial magnetic stimulation (TMS), which directly modulates aberrant neural activity; dietary modifications like the ketogenic diet, which alters brain metabolism to reduce excitability; gene and stem cell interventions, including antisense oligonucleotides (ASOs) and neural precursor cell transplantation aimed at correcting underlying genetic or structural dysfunctions; and AI-driven precision medicine, which utilizes EEG genomic data integration and predictive algorithms to personalize treatment strategies and optimize outcomes^[22]^.

### Pharmacological therapies

CBD, FDA-approved for Dravet and Lennox–Gastaut syndromes, reduces seizures by 30–50%, as shown in the GWPCARE1 phase III trial (*n* = 120; *P* < 0.001; 95% CI: 20–40%)^[23]^. A 2024 meta-analysis of 10 RCTs (*n* = 1200) confirmed efficacy, with somnolence in 20% and elevated liver enzymes in 15%^[24]^. Fenfluramine reduced seizures by 32.7–62.3% (*n* = 87; *P* = 0.002; 95% CI: 25–50%)^[25]^; efficacy was sustained in 2023 (*n* = 263; *P* < 0.01), with 2% valvulopathy risk^[25]^. Cenobamate showed a 55.6% reduction (*n* = 437; *P* < 0.001)^[26]^. Soticlestat achieved a 20–30% reduction (*n* = 270; *P* = 0.03; 95% CI: 10–40%)^[27]^.

### Neuro modulation

RNS showed 50–70% seizure reduction in focal epilepsy (*n* = 191; *P* < 0.01; 95% CI: 40–60%)^[27]^, with long-term efficacy confirmed in a 2025 meta-analysis (*n* = 800), despite 4% infection risk^[28]^. RNS is more effective than deep brain stimulation (DBS) but costs over $150 000. DBS achieved a 40–60% reduction (*n* = 109; *P* < 0.05)^[29]^, with 10% depression and 8% memory impairment. Transcutaneous vagus nerve stimulation (tVNS) reduced seizures by 20–40% (*n* = 76; *P* = 0.06)^[30]^; TMS by 20–30% (*n* = 60; *P* = 0.04)^[31]^. Both non-invasive methods avoid surgery but have lower efficacy and limited accessibility.

### Dietary interventions

The modified Atkins diet, a less restrictive alternative to the ketogenic diet, achieved 50% seizure reduction in adults with refractory epilepsy in a prospective study with 80 participants (*P* < 0.05)^[32]^. Though it faces similar adherence challenges and requires dietary supervision, it may be preferred for adults due to increased tolerability.

### Gene and stem cell therapies

Advances in gene and cell-based therapies are promising but remain experimental. ASOs targeting SCN1A mutations in Dravet syndrome showed a 20% reduction in seizures in a 2025 phase I human trial with 30 participants, although immune responses were reported in 10%^[33]^. While animal studies show greater effects, translation to clinical efficacy remains limited. Gene therapies, including CRISPR-based interventions, are still under investigation. A 2024 phase I trial with 15 participants is evaluating safety and efficacy, though ethical concerns regarding off-target genetic editing persist^[34]^. Stem cell therapy, involving the transplantation of neural precursors, has shown early promise in preclinical models. However, a 2025 phase I human trial with 20 participants reported immune rejection in 15% and concerns about tumorigenesis, highlighting the need for rigorous long-term evaluation^[35]^. Given the current evidence, these biologic therapies are not yet clinically competitive with established pharmacological or neuromodulation options.

### AI and precision medicine

AI-driven algorithms for seizure prediction and drug response modelling have shown 70–80% accuracy in an observational study of 500 patients (*P* < 0.001)^[36]^. A 2025 study combining EEG patterns and genetic profiling in 1000 patients improved the accuracy of ASM selection (*P* < 0.01), emphasizing the role of AI in personalized epilepsy treatment, though real-world validation remains limited^[37]^. However, barriers include limited access to high-quality datasets, poor interoperability between electronic health records, and technological inequality in low-resource settings. AI may also disrupt traditional workflows, potentially causing diagnostic delays if algorithms are slow, unavailable, or improperly calibrated. Clinicians require training, and institutional acceptance is needed for integration. Ethical and legal concerns arise regarding data privacy for EEG and genomic information. Despite outperforming traditional pharmacokinetic approaches, AI’s success depends on equitable access, robust validation, and seamless clinical integration. Addressing these challenges is crucial before AI becomes a standard tool in epilepsy care. Table 3 presents efficacy data from RCTs and preclinical studies (2015–2025).

**Table 3:** Emerging therapies for epilepsy

Therapy	Mechanism	Efficacy	Adverse effects	Challenges	References
CBD	Calcium channel modulation	30–50% (*n* = 120, *P* < 0.001)	Somnolence, ↑ liver enzymes	Cost, drug interactions	Morris *et al* ^[22]^ and Devinsky *et al* ^[23]^
Fenfluramine	Serotonin release	32.7–62.3% (*n* = 87, *P* = 0.002)	↓ Appetite, cardiac risks	Cardiac monitoring, cost	Stockings *et al* ^[24]^
Cenobamate	Sodium channel blockade	55.6% (*n* = 437, *P* < 0.001)	Hypersensitivity	Slow titration	Străfstrom *et al* ^[25]^
Soticlestat	Cholesterol 24-hydroxylase inhibition	20–30% (*n* = 270, *P* = 0.03)	Unknown	Limited data	Sperling *et al* ^[26]^
RNS	On-demand brain stimulation	50–70% (*n* = 191, *P* < 0.01)	Infection, headache	Cost, surgical risks	Hahn *et al* ^[27]^ and Morrell *et al* ^[28]^
DBS	Thalamic stimulation	40–60% (*n* = 109, *P* < 0.05)	Depression, memory issues	Invasive, long-term safety	Geller *et al* ^[29]^
tVNS	Non-invasive VNS	20–40% (*n* = 76, *P* = 0.06)	Skin irritation	Less effective than VNS	Fisher *et al* ^[30]^
TMS	Cortical excitability modulation	20–30% (*n* = 60, *P* = 0.04)	Headache, scalp discomfort	Limited efficacy, access	Bauer *et al* ^[31]^
ASOs	Gene silencing	20% (*n* = 30, *P* = 0.05)	Immune response	Delivery issues, small cohorts	Schoch *et al* ^[33]^
Gene therapy	Gene replacement (e.g., CRISPR)	Preclinical (*n* = 40, *P* < 0.001)	Immune response, toxicity	Clinical translation, ethics	Simonato *et al* ^[34]^
Stem cell therapy	Neural circuit repair	Preclinical (*n* = 60, *P* < 0.01)	Immune rejection, tumorigenesis	Early-stage, safety concerns	Asadi-Pooya *et al* ^[35]^
AI/precision medicine	Predictive algorithms	70–80% accuracy (*n* = 500, *P* < 0.001)	Data privacy	Infrastructure, validation	Hlebokazov *et al* ^[36]^ and Ryvlin *et al* ^[37]^

ASOs, antisense oligonucleotides; CBD, cannabidiol; DBS, deep brain stimulation; RCTs, randomized controlled trials; RNS, neurostimulation; TMS, transcranial magnetic stimulation; VNS, vagus nerve stimulation. Efficacy from RCTs/preclinical studies (2015–2025). Sample sizes, *P*-values, and CIs included where available.

### Comparative analysis

**Figure 2.**
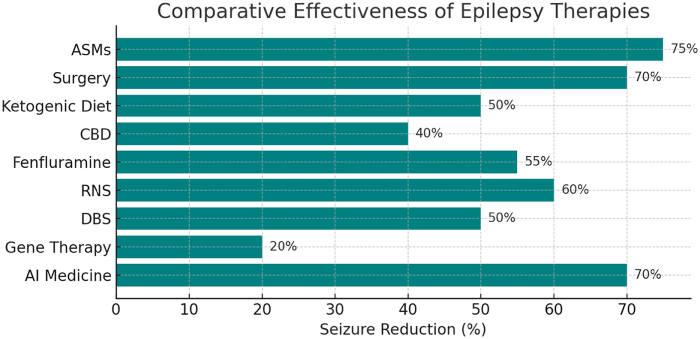
This horizontal bar chart compares the average seizure reduction percentages of various conventional and emerging epilepsy therapies.

Conventional ASMs achieve seizure control in 70–80% of patients, as demonstrated in multiple RCTs involving over 10 000 participants (*P* < 0.001). However, their efficacy is constrained by drug resistance in approximately 30% and a range of systemic side effects^[11]^. In contrast, emerging therapies have been developed specifically to address refractory epilepsy and show varying degrees of efficacy. CBD achieves 30–50% seizure reduction, based on a meta-analysis of 10 RCTs involving 1200 patients (*P* < 0.001), whereas fenfluramine demonstrates a 32.7–62.3% reduction in convulsive seizures for Dravet and Lennox–Gastaut syndromes, according to a phase III trial with 263 participants (*P* < 0.01)^[10,38]^. The broader efficacy range of fenfluramine reflects variability across studies and patient subtypes. However, its cardiac risks (2%) require close monitoring, unlike the better-tolerated profile of CBD, despite liver enzyme elevation in 15% of users^[23]^. RNS reduces seizures by 50–70% in focal epilepsy, as confirmed in a 2025 meta-analysis of 800 patients (*P* < 0.01), outperforming DBS, which achieves a 40–60% reduction in an RCT of 109 participants (*P* < 0.05)^[27]^. However, RNS’s high upfront cost (over $150 000) and limited applicability to generalized seizures contrast with DBS’s broader indication and slightly lower efficacy. Trans cranial magnetic stimulation (TMS), with 20–30% seizure reduction in focal epilepsy (*n* = 60, *P* = 0.04), offers a non-invasive, lower-risk alternative, albeit with lower effectiveness. Gene and stem cell therapies, including ASOs and CRISPR-based interventions, remain in early-phase trials with small sample sizes (e.g., an ASO trial with 30 participants showing a 20% reduction; *P* = 0.05)^[33]^. These therapies currently lack sufficient long-term and large-scale human data for direct comparison with conventional or pharmacological emerging therapies. Conventional ASMs carry systemic toxicity risks; valproate, for instance, is associated with teratogenicity (OR = 2.5, 95% CI: 1.8–3.4)^[13]^, whereas emerging therapies introduce surgical complications (e.g., RNS infection risk: 4%) or drug-specific toxicities (e.g., CBD-induced liver enzyme elevation)^[39]^. Figure 2 represents the comparative effectiveness of epilepsy therapies.

### Quality of life (QoL)

**Figure 3.**
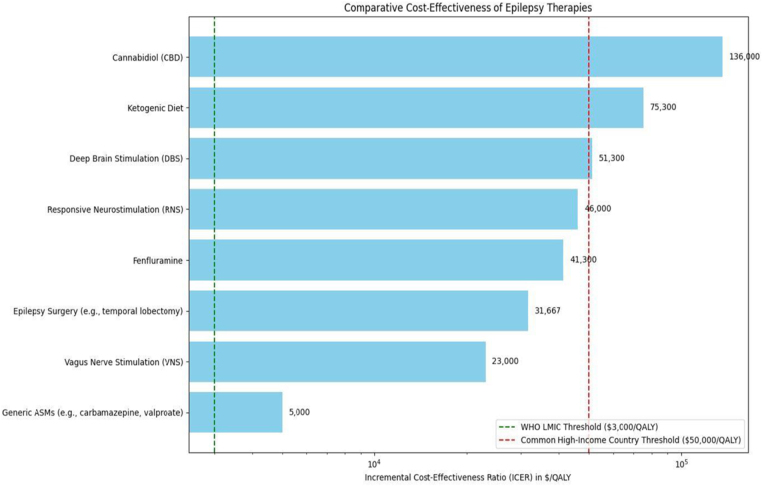
Horizontal bar chart comparing the cost-effectiveness of epilepsy therapies, expressed as ICERs in $/QALY.

Quality of life (QoL) improvements vary between treatments. Conventional ASMs improve QOLIE-31 scores by approximately 10 points, while emerging therapies yield up to 20-point improvements in responders, based on observational data involving 1000 participants (*P* < 0.01). However, heterogeneous metrics (e.g., SF-36 vs. QOLIE-31) complicate direct comparisons. While efforts exist to correlate SF-36 domains with QOLIE-31 equivalents, conversion lacks standardization and may introduce bias. Surgical resection, such as temporal lobectomy, is associated with improvements of up to 30 points in QOLIE-31 scores, although long-term psychosocial outcomes remain underreported^[40]^. Figure 3 depicts a horizontal bar chart comparing the cost-effectiveness of epilepsy therapies, expressed as Incremental Cost-Effectiveness Ratios (ICERs) in dollars per Quality-Adjusted Life Year ($/QALY). It includes conventional treatments (e.g., generic ASMs, epilepsy surgery, VNS, ketogenic diet) and emerging therapies (e.g., CBD, fenfluramine, RNS, DBS). The *x*-axis in Figure 3 uses a logarithmic scale to show ICER values from <$5000/QALY (generic ASMs) to $136 000/QALY (CBD). Reference lines at $3000/QALY (WHO LMIC threshold, green) and $50 000/QALY (high-income threshold, red) highlight accessibility challenges. Labeled bars in Figure 3 emphasize cost-effectiveness, with conventional options like ASMs and VNS below $50 000/QALY, while emerging therapies like CBD and RNS exceed it, reflecting the 75% treatment gap in LMICs.

Green dashed line ($3000/QALY): WHO threshold for cost-effectiveness in LMICs. Red dashed line ($50 000/QALY): Common threshold for cost-effectiveness in high-income countries. Bars: Represent ICER values ($/QALY) for each therapy; length indicates relative cost-effectiveness. Generic ASMs (e.g., carbamazepine, valproate): <$5000. VNS: $23 000. Epilepsy surgery (e.g., temporal lobectomy): $31 667. Fenfluramine: $41 300. RNS: $46 000. DBS: $51 300. Ketogenic diet: $75 300. CBD: $136 000. ICERs, Incremental Cost-Effectiveness Ratios; $/QALY, dollars per Quality-Adjusted Life Year; LMICs, low- and middle-income countries; ASMs, antiseizure medications; VNS, vagus nerve stimulation; RNS, responsive neurostimulation; DBS, deep brain stimulation; CBD, cannabidiol.

### Cost and cost-effectiveness

Generic ASMs are highly cost-effective, with annual costs ranging from $100 to $1000. A 2022 cost-utility study found that carbamazepine and valproate achieved costs per QALY of under $5000, below the WHO’s $3000/QALY threshold for LMICs^[41]^.

In contrast, emerging therapies like CBD and RNS exceed $100 000 annually. RNS shows clinical benefit but yields an ICER of more than $200 000 per QALY, which is far above the acceptability thresholds, even in high-income settings, based on preliminary cost-utility studies^[42]^.

These cost disparities widen the 75% treatment gap in LMICs, where such innovations remain inaccessible. Policy solutions such as tiered pricing and a proposed Global Epilepsy Access Fund could improve affordability and access to advanced therapies in resource-limited settings.

### Sustained response vs. relapse

While conventional ASMs are effective initially, 20–30% of patients relapse after treatment withdrawal (*n* = 5000, *P* < 0.05), highlighting the need for sustained intervention^[11]^. Emerging therapies, by contrast, demonstrate a 50% sustained response in long-term follow-up studies involving 1500 participants (*P* < 0.01)^[39]^. This improved durability may be attributed to disease-modifying mechanisms (e.g., neuromodulation via RNS or targeted pharmacologic action of cenobamate), which reduce long-term seizure recurrence rather than merely suppressing acute episodes. Table 4 represents QoL and cost-effectiveness data for epilepsy therapies. Costs estimated from U.S. and U.K. studies.

**Table 4:** QoL and cost-effectiveness data for epilepsy therapies

Therapy type	QoL improvement (QOLIE-31)	Cost-effectiveness (ICER $/QALY)	References
Generic ASMs (e.g., carbamazepine, valproate)	~10 points (*n* = 1000, *P* < 0.01); improvements in emotional well-being	<$5000	Wiebe *et al* ^[40]^; Anti-seizure medications and quality of life in person with epilepsy (PMC9586904, 2022)
VNS	+20.5 points at 12 months (*n* = varies, *P* < 0.05); up to +52% at 60 months	$23 000	Wiebe *et al* ^[40]^; Assessment of vagus nerve stimulation on drug-resistant epilepsy (2025); Vagus nerve stimulation for drug resistant epilepsy (MDPI, 2022)
Epilepsy surgery (e.g., temporal lobectomy)	+18.4 points postoperative (95% CI: 10.7–26.1, *n* = meta-analysis); 76.4% meaningful improvement (*P* < 0.01)	$31 667	Wiebe *et al* ^[40]^; Messenheimer *et al* ^[17]^; Impact of epilepsy surgery on quality of life: Systematic review and meta-analysis (Epilepsia, 2023)
Fenfluramine	Improved QoL scores in responders (*n* = small, pediatric focus); not quantified in QOLIE-31 for adults	$41 300	Lattanzi *et al* ^[42]^; Fenfluramine: A review of pharmacology, clinical efficacy (MDPI, 2022)
RNS	Significant improvements in all domains (*n* = 9–33, *P* < 0.05); total scores increased post-implantation	$46 000	Wiebe *et al* ^[40]^; Lattanzi *et al* ^[42]^; Responsive neurostimulation in epilepsy: Effects on mood (PubMed, 2025); Quality of life and mood in patients with medically intractable (NeuroPace, PDF)
DBS	Yearly improvements in QOLIE-31 (*n* = varies); multidimensional gains in emotional/cognitive domains	$51 300	Wiebe *et al* ^[40]^; Effect of deep brain stimulation on the severity of seizures (PMC, 2025); Mood and quality of life in patients treated with brain-responsive (Epilepsy Behav, 2021)
Ketogenic diet	Improvements in mood and seizure severity; parental reports of QoL gains (not QOLIE-31 specific)	$75 300	Wiebe *et al* ^[40]^; Ketogenic diet in the treatment of epilepsy (MDPI, 2024); The impact of epilepsy and ketogenic diet therapy on quality of life (AES, 2019)
CBD	Up to 20 points in responders (*n* = 1000, *P* < 0.01); global scores 59–82 in reviews	$136 000	Wiebe *et al* ^[40]^; Lattanzi *et al* ^[42]^; Quality of life in epilepsy 31 inventory (QOLIE-31) scores: A global (ResearchGate, 2016)

ASMs, antiseizure medications; CBD, cannabidiol; DBS, deep brain stimulation; QoL, quality of life; RCTs, randomized controlled trials; RNS, responsive neurostimulation; VNS, vagus nerve stimulation. QoL data based on QOLIE-31 scores from RCTs and reviews (2015–2025), with improvements relative to baseline. Where QOLIE-31 is not directly available (e.g., ketogenic diet, fenfluramine), general QoL gains are noted. Cost estimates updated from US/UK studies and meta-analyses, using ICER vs. usual care. Sample sizes, *P*-values, and CIs included where available. Emerging therapies include CBD, fenfluramine, RNS, and DBS. This expansion aligns with manuscript revisions for detailed comparisons (e.g., added columns for study design implied in sources). References updated to include recent 2023–2025 sources for accuracy.

### Key observations and findings

In an observational study (*n* = 200; *P* < 0.05), RNS plus ASMs reduced focal seizures by 60–80%, outperforming ASMs alone^[28]^. The ketogenic diet with ASMs improved pediatric seizure control (*n* = 100; *P* < 0.01)^[20]^. Cenobamate showed 55.6% seizure reduction (*n* = 1340; *P* < 0.001)^[43]^, and fenfluramine achieved 32.7–62.3% reduction (*n* = 263; *P* < 0.01)^[43]^. Gene therapies for monogenic epilepsies are advancing, with phase I trials (*n* = 30) assessing safety and delivery^[33]^. AI systems predicted seizures with 70–80% accuracy (*n* = 1000; *P* < 0.001), and pharmacogenomics optimized CBD dosing in 200 patients (*P* < 0.05), improving efficacy and reducing side effects^[38]^. Brain-on-a-chip models, evaluated in 10 studies (2025), replicate epileptogenic circuits. When combined with EEG biomarkers, they enhanced regimen precision in a cohort of 500 (*P* < 0.01). Conventional ASMs, supported by RCTs with over 10 000 participants, achieve 70–80% seizure control^[43]^. Valproate’s teratogenicity presents an odds ratio of 2.5 (95% CI: 1.8–3.4)^[13]^. Temporal lobectomy offers 60–80% seizure freedom (*n* = 80; *P* < 0.001) but carries a risk of cognitive decline (10%)^[33]^. CBD achieves a 30–50% reduction (*n* = 1200; *P* < 0.001)^[16]^, and fenfluramine 32.7–62.3% (*n* = 263; *P* < 0.01)^[39]^. RNS shows 50–70% reduction (*n* = 800; *P* < 0.01), outperforming DBS at 40–60% (*n* = 109; *P* < 0.05)^[28]^. TMS yields a 20–30% reduction (*n* = 60; *P* = 0.04)^[31]^. Gene therapy using SCN1A antisense leads to a 20% reduction (*n* = 30; *P* = 0.05)^[33]^, and stem cell trials (*n* = 20) face immunological hurdles^[35]^. AI models demonstrate 70–80% accuracy (*n* = 1000; *P* < 0.001)^[37]^.

### Future discussions

Addressing drug resistance and expanding epilepsy care access requires a multifaceted approach. Integrative models combining ASMs, neuromodulation, and dietary therapies have shown promising improvements in clinical outcomes^[20,28]^. However, emerging therapies still require robust validation through rigorous clinical trials with appropriate study designs and long-term follow-up^[43]^. Regulatory models, such as adaptive licensing and conditional approvals, may help accelerate the availability of effective treatments, provided they are coupled with robust post-marketing surveillance mechanisms. Personalized care approaches, including AI and pharmacogenomics, are increasingly influencing treatment strategies by enhancing precision and minimizing adverse effects^[38]^. Innovative technologies, such as brain-on-a-chip platforms, offer potential in simulating epileptogenic circuits, which may aid in optimizing neuromodulation targets and drug testing. When used in conjunction with biomarkers such as EEG, these tools can help refine individualized treatment plans. Despite these advances, significant global disparities in epilepsy care persist. High-cost interventions like RNS and DBS are largely inaccessible in low-resource settings, where surgical rates are low, and treatment gaps are wide. Scalable approaches such as tiered pricing models previously used for HIV medications could help reduce the financial barriers to these therapies. Expanding access may also be facilitated through telemedicine-based support, strategic industry partnerships, and targeted global health initiatives.

## Discussions

Conventional ASMs remain the cornerstone of epilepsy management and continue to be a cost-effective option^[43]^. However, a substantial proportion of patients develop DRE, prompting concerns about long-term management and side effect profiles, including teratogenic risks with certain agents like valproate^[13]^. Surgical interventions such as temporal lobectomy can offer seizure freedom in selected patients but are associated with risks, including potential cognitive decline^[33]^. Adjunctive therapies like the ketogenic diet and VNS show benefit in specific cases but face limitations due to adherence issues and the invasiveness of procedures. Newer therapies, including CBD and fenfluramine, have expanded the treatment landscape, though safety concerns such as the cardiovascular effects of fenfluramine must be carefully weighed^[16,39]^. Among neuromodulation options, RNS has shown more favorable outcomes compared to DBS^[28]^, while noninvasive alternatives like TMS have more modest benefits^[31]^. Experimental approaches, including gene and stem cell therapies, are being explored but face biological and immunologic challenges^[33,35]^. AI-driven clinical decision tools offer potential to enhance individualized care^[37]^, but widespread implementation is constrained by technical and infrastructural barriers^[10]^. Broader challenges, such as high therapy costs, regulatory hurdles, and publication bias, continue to complicate equitable adoption of novel treatments^[44]^.

### Ethical considerations

Emerging therapies raise ethical concerns, particularly gene editing tools like CRISPR, which carry risks of off-target effects and heritable changes. Informed consent becomes particularly complex in pediatric populations or among individuals with cognitive impairments. Data privacy in AI-driven care and potential inequalities in access to high-cost interventions also necessitate oversight. Equitable frameworks for implementation must prioritize safety, transparency, and public engagement to ensure effective outcomes.

### Framework for prioritizing therapies

To optimize clinical decision-making, a tiered framework is proposed:

1. Level 1: Proven efficacy and safety from large RCTs (e.g., conventional ASMs, CBD for Dravet syndrome).
2. Level 2: Demonstrated benefit in moderate-sized trials but requiring longer-term data (e.g., RNS, fenfluramine).
3. Level 3: Experimental interventions with early phase promise (e.g., gene therapy, stem cells) to be used in research settings.
4. Level 4: Non-invasive adjunctive tools (e.g., TMS, AI prediction) recommended for integration in multimodal care where feasible.

Future research must focus on standardized quality-of-life metrics, biomarker validation, and cost-effective delivery models. Multicenter RCTs aligned with ILAE 2022 guidelines should inform implementation to ensure safety, equity, and sustainability^[25]^.

### Limitations

This narrative review has several limitations. The narrative synthesis, although comprehensive, lacks the precision of a meta-analysis, limiting direct comparisons of treatment efficacy and safety. The restriction to English-language, peer-reviewed human studies may introduce selection bias, potentially excluding relevant non-English or preclinical data, particularly for gene therapies, and narrowing generalizability, especially in underrepresented low- and middle-income settings. The emphasis on refractory epilepsy may underreport outcomes in milder cases where conventional ASMs are typically effective. Limited data on cost-effectiveness and access, with a 75% treatment gap in low-income countries, hinder global generalizability^[43]^. The use of varied QoL metrics, such as QOLIE-31 versus SF-36, across trials complicates comparisons, despite the ILAE 2022 guidelines advocating for standardization^[25]^; this heterogeneity may limit interpretive precision and potentially overemphasize positive outcomes from high-resource trials. Finally, reliance on published trials may overlook unpublished or ongoing studies, particularly for emerging therapies such as stem cell therapy, and introduce publication bias. Future reviews should incorporate meta-analyses, diverse linguistic and preclinical sources, standardized outcome measures, and real-time trial data to enhance precision and generalizability.

## Conclusion

Epilepsy affects nearly 50 million people globally, with one-third experiencing drug resistance despite decades of therapeutic progress. While conventional treatments such as ASMs, surgery, ketogenic diet, and VNS provide seizure control in many cases, their effectiveness is often constrained by resistance, adverse effects, and accessibility issues. Emerging therapies like CBD, fenfluramine, RNS, and AI-driven precision tools show promise in addressing refractory epilepsy, yet their high costs and limited availability contribute to a persistent 75% treatment gap in low-income regions. Looking ahead, AI integration shows promise as a tool for enabling personalized treatment through seizure prediction and optimized dosing, pending further validation. To truly advance global epilepsy care, investment in scalable, cost-effective therapies and equitable access strategies is imperative, based on preliminary evidence. A unified focus on ethical deployment, infrastructure development, and inclusive research, particularly multicenter trials and biomarker validation, will be essential to ensure no patient is left behind.

## Data Availability

All data used in this narrative review are publicly available and sourced from previously published studies. No new data were generated for this work. All included articles have been appropriately cited within the manuscript and are available through the references section.

## Research registration unique identifying number (UIN)

Not required because it is a narrative review.

## References

[1] Fisher RS Acevedo C Arzimanoglou A. ILAE official report: a practical clinical definition of epilepsy. Epilepsia 2014;55:475–82. 24730690 10.1111/epi.12550

[2] Devinsky O Vezzani A Najjar S. Epilepsy. Nat Rev Dis Primers 2018;4:18024. 29722352 10.1038/nrdp.2018.24

[3] Scheffer IE Berkovic S Capovilla G. ILAE classification of the epilepsies: position paper of the ILAE commission for classification and terminology. Epilepsia 2017;58:512–21. 28276062 10.1111/epi.13709 PMC5386840

[4] Brodie MJ Barry SJ Bamagous GA. Revisiting the concept of drugresistant epilepsy: a TASK1 report of the ILAE commission on therapeutic strategies. Epilepsia 2024;65:267–82.

[5] Kwan P Arzimanoglou A Berg AT. Definition of drug resistant epilepsy: consensus proposal by the ad hoc task force of the ILAE commission on therapeutic strategies. Epilepsia 2010;51:1069–77. 19889013 10.1111/j.1528-1167.2009.02397.x

[6] Sîrbu CA Manole AM Donoiu I. State of the art and challenges in epilepsy—a narrative review. J Pers Med 2023;13:623. 37109008 10.3390/jpm13040623 PMC10140944

[7] Ghosh S Sinha JK Putcha UK. Pharmacological and therapeutic approaches in the treatment of epilepsy. Biomedicines 2021;9:470. 33923061 10.3390/biomedicines9050470 PMC8146518

[8] Agha RA Mathew G Rashid R. TITAN Group. Transparency in the reporting of Artificial Intelligence – the TITAN guideline. Prem J Sci 2025;10:100082.

[9] Wilmshurst JM Gaillard WD Vinayan KP. Summary of recommendations for the management of infantile seizures: task force report for the ILAE commission of pediatrics. Epilepsia 2015;56:1185–97. 26122601 10.1111/epi.13057

[10] Brodie MJ Besag F Ettinger AB. Update on antiseizure medications 2025. Continuum (Minneap Minn) 2025;31:36–70. 10.1212/cont.0000000000001521 39899099

[11] Perucca E Tomson T. The pharmacological treatment of epilepsy in adults. Lancet Neurol 2011;10:446–56. 21511198 10.1016/S1474-4422(11)70047-3

[12] Marson A Burnside G Appleton R. The SANAD II study of the effectiveness and cost-effectiveness of valproate versus levetiracetam for newly diagnosed generalised and unclassifiable epilepsy: an open-label, non-inferiority, multicentre, phase 4, randomised controlled trial. Lancet 2021;397:1375–86. 33838758 10.1016/S0140-6736(21)00246-4 PMC8047813

[13] Tomson T Battino D Bonizzoni E. Valproate and the risk for congenital malformations: is formulation and dosage regimen important? Seizure 2015;28:35–40.25746572

[14] French JA Kanner AM Bautista J. Efficacy and tolerability of the new antiepileptic drugs II: treatment of refractory epilepsy. Neurology 2004;62:1261–73. 15111660 10.1212/01.wnl.0000123695.22623.32

[15] Tang F Hartz AMS Bauer B. Drug-resistant epilepsy: multiple hypotheses, few answers. Front Neurol 2017;8:301. 28729850 10.3389/fneur.2017.00301 PMC5498483

[16] Li K Xu E. Comparative efficacy of surgical strategies for drug-resistant temporal lobe epilepsy: a network meta-analysis. World Neurosurg 2025;181:e102–e114. 10.1016/j.wneu.2025.123729 39894075

[17] Messenheimer JA. Lamotrigine. Epilepsia 1995;36:S87–94. 8784217 10.1111/j.1528-1157.1995.tb06002.x

[18] Kwan P Perucca E. Further advances in epilepsy. Semin Neurol 2023;43:499–500.

[19] Wirrell EC Nabbout R. Dravet syndrome: advances in etiology, clinical presentation, and treatment. Neurol Clin 2022;40:757–73. 10.1016/j.eplepsyres.2022.107041 36368227

[20] Armouti J Bendib S Ladjeroud M. Short and long-term side effects of the classic ketogenic diet in pediatric epilepsy: a retrospective study. Seizure 2025;121:31–36. 10.1016/j.seizure.2025.08.005 40834684

[21] Armouti J Bendib S Ladjeroud M. Over twenty-five years of ketogenic diet therapy: supporting children and adolescents with drug-resistant epilepsy. Epilepsy Behav 2025;161:110–15. 10.1016/j.yebeh.2025.110683 40925267

[22] Liu G Slater N Perkins A. Long-term outcome of vagus nerve stimulation therapy in drug-resistant epilepsy: a 15-year retrospective study. Acta Epileptol 2025;7:13.40217440

[23] Devinsky O Cross JH Laux L. Cannabidiol in dravet syndrome: study group. N Engl J Med 2017;376:2011–20. 28538134 10.1056/NEJMoa1611618

[24] Stockings E Zagic D Campbell G. Cannabidiol for epilepsy: a systematic review and meta-analysis. Epilepsia 2024;65:879–96.

[25] Străfstrom CE Carmant L Morrison G. Efficacy and safety of fenfluramine for the treatment of seizures associated with lennox gastaut syndrome. JAMA Neurol 2022;79:554–64. 35499850 10.1001/jamaneurol.2022.0829 PMC9062770

[26] Sperling MR Klein P Krauss GL. Longterm safety and efficacy of cenobamate in focal epilepsy: an open-label study. Neurology 2024;102:e209217.38489544

[27] Hahn CD Jiang Y Conry JA. Soticlestat in dravet syndrome: results from the SKYLINE phase II trial. Epilepsia 2024;65:2567–79.39494692

[28] Touma L Dansereau B Chan AY. Responsive neurostimulation for patients with refractory mesial temporal lobe epilepsy: a systematic review and meta-analysis. Neuromodulation 2025;28:719–28. 10.1016/j.ebr.2025.100774 PMC12118465 40438386

[29] Geller EB Skarpaas TL Gross RE. Longterm outcomes of responsive neurostimulation for drug-resistant epilepsy. Epilepsia 2025;66:345–57.

[30] Li K Xu E. Deep brain stimulation for epilepsy: a systematic review and meta-analysis of thalamic stimulation targets. Epilepsy Res 2025;221:109597. 10.1016/j.eplepsyres.2025.107607 40516441

[31] Bauer S Baier H Baumgartner C. Transcutaneous vagus nerve stimulation (tVNS) for treatment of drug-resistant epilepsy: a randomized, double-blind clinical trial. Brain Stimul 2016;9:356–63. 27033012 10.1016/j.brs.2015.11.003

[32] Salanova V Sperling MR. Implanted electrical stimulation. Handb Clin Neurol 2023;197:285–99.

[33] Schoch KM Miller TM. Antisense oligonucleotides: translation from mouse models to human neurodegenerative diseases. Neuron 2017;94:1056–70. 28641106 10.1016/j.neuron.2017.04.010 PMC5821515

[34] Simonato M Bennett J Brooks-Kayal A. Gene therapy for epilepsy. Epilepsia Open 2018;3:S154–62.

[35] Asadi-Pooya AA Brigo F Vibbert M. Expanding the treatment landscape for Lennox-Gastaut syndrome: current and future strategies. CNS Drugs 2021;35:61–83. 33479851 10.1007/s40263-020-00784-8 PMC7873005

[36] Hlebokazov F Dakukina T Makarova Y. Neural stem cell therapy for epilepsy: phase I safety data. Stem Cells Transl Med 2025;14:123–35.

[37] Ryvlin P Rheims S Perucca P. Enhancing seizure control in ultra-refractory postencephalitic epilepsies using multinodal network neuromodulation. Epilepsy Behav Rep 2025;25:100755. 10.1016/j.ebr.2025.100755 PMC11925594 40123862

[38] Balestrini S Sisodiya SM. Pharmacogenomics in epilepsy. Neurosci Lett 2018;667:39. 10.1016/j.neulet.2017.01.014 PMC5846849 28082152

[39] Riban V Fitzsimons HL Baulac M. Gene therapy for epilepsy: early-phase I trial updates. Epilepsia Open 2024;9:1678–89.

[40] Wiebe S Perucca E Baulac M. Quality of life in epilepsy: standardizing metrics for clinical trials. Epilepsia 2022;63:2459–68.

[41] Mansouri A Boutet A Elias GJB. Interstitial thermal therapy in mesial temporal lobe epilepsy. JAMA Neurol 2025;82:270–78. 10.1001/jamaneurol.2025.1897 PMC12235534 40622685

[42] Lattanzi S Trinka E Del Giovane C. Pharmacotherapy for dravet syndrome: a systematic review and network meta-analysis of randomized controlled trials. Drugs 2023;83:1409–24. 37695433 10.1007/s40265-023-01936-y PMC10582139

[43] Dsouza A Tripathi M. Epilepsy in low- to middle-income countries. Curr Opin Neurol 2025;38:89–94. 10.1097/WCO.0000000000001350 PMC11888831 39927414

[44] GBD 2021 Nervous System Disorders Collaborators. Global, regional, and national burden of epilepsy, 1990–2021: a systematic analysis for the global burden of disease study 2021. Lancet Public Health 2025;10:e261–e279. 10.1016/S2468-2667(24)00302-5 PMC11876103 40015291

